# Oxycodone Alleviates Endometrial Injury via the TLR4/NF-*κ*B Pathway

**DOI:** 10.1155/2022/6153279

**Published:** 2022-02-23

**Authors:** Aibing Zhu, Jianping Yang

**Affiliations:** ^1^Department of Anesthesiology, The First Affiliated Hospital of Soochow University, Suzhou 215006, China; ^2^Department of Anesthesiology, The Affiliated Wuxi Matemity and Child Health Care Hospital of Nanjing Medical University, Wuxi 214002, China

## Abstract

Endometrial injury is a common female disease. This study was designed to illustrate the effects of oxycodone on mifepristone-induced human endometrial stromal cells (hEndoSCs) injury and delineate the underlying molecular mechanism. hEndoSCs were stimulated with mifepristone to generate the endometrial injury in vitro model. hEndoSCs viability, cytotoxicity, and apoptosis were measured by methyl thiazolyl tetrazolium (MTT) assay, lactate dehydrogenase assay (LDH), and flow cytometry (FCM) analysis, respectively. Meanwhile, quantitative reverse transcription polymerase chain reaction (RT-qPCR) and Western blot assay were conducted to evaluate gene and protein expressions. The secretions of inflammatory cytokines (TNF-*α*, IL-1*β*, and IL-6) were measured using enzyme-linked immunosorbent assay (ELISA). The data revealed that mifepristone exposure memorably inhibited hEndoSCs viability and promoted cell apoptosis and inflammatory cytokines secretion, and oxycodone had no cytotoxicity on hEndoSCs. Oxycodone increased hEndoSCs growth, blocked cell apoptosis, enhanced Bcl-2 expression, reduced Bax levels, and decreased the secretion of inflammatory cytokines in mifepristone-induced hEndoSCs, exhibiting the protective effects in endometrial injury. In addition, the TLR4/NF-*κ*B pathway-related protein levels (TLR4 and p-p65) in mifepristone-treated hEndoSCs were enhanced, while these enhancements were inhibited by oxycodone treatment. In conclusion, oxycodone exhibited the protective role in mifepristone-triggered endometrial injury via inhibiting the TLR4/NF-*κ*B signal pathway.

## 1. Introduction

The endometrium is a layer of the inner wall of the uterus, with the change of women's physiological cycle, the endometrium will also change, and it has a great impact on menstruation and fertility [1, 2]. The main symptoms of endometrial damage are menstrual disorders, dysmenorrhea, and endometriosis [3]. Endometrial damage and adhesion will result in the obstruction of menstrual blood discharge, thus causing dysmenorrhea and infection, and under the stimulation of pelvic inflammatory disease, leads to endometriosis and ultimately affect pregnancy [4, 5].

Oxycodone, a semisynthetic opioid extracted from alkaloid tibain, has been used as a potent analgesic for more than 80 years [6]. Oxycodone is widely used in clinics because of its high bioavailability and multiple routes of administration [7, 8]. Intravenous oxycodone can be safely used in painless ovum retrieval without increasing respiratory depression, prolonging recovery time, and reducing the dosage of propofol and postoperative pain [9]. To the best of our knowledge, whether oxycodone has a protective effect on endometrial injury and its molecular mechanism are still unclear.

Accumulating evidences have demonstrated that multiple factors including ROS/NLRP3/caspase-1/GSDMD, WNT, and mTOR signaling pathway were involved in the progression of endometrial diseases [10–12]. The TLR4/NF-*κ*B pathway has been identified to be involved in many diseases. Bai et al. suggested that biochanin A attenuates myocardial ischemia/reperfusion injury through the TLR4/NF-*κ*B/NLRP3 signaling pathway [13]. Also, Zhou et al. revealed that galectin-3 activates TLR4/NF-*κ*B signaling to promote lung adenocarcinoma cell proliferation through activating lncRNA-NEAT1 expression [14]. However, whether the TLR4/NF-*κ*B signaling pathway is involved in the development of endometrial injury needs to be further explored.

Mifepristone is a very active human antiprogesterone and antiglucocorticoid drug [15]. Studies have shown that mifepristone can directly damage the endometrium in vitro and in vivo [16, 17]. The prevention and reversal of endometrial injury caused by mifepristone is a clinical challenge. The human endometrium is a complex tissue, mainly composed of human endometrial stromal cells (hEndoSCs) which play an essential role in maintaining endometrial homeostasis, and oxidative stress of hEndoSCs is responsible for preeclampsia, decidualization failure, and endometritis [18, 19]. Recently, mifepristone-induced hEndoSCs injury has been used to study endometrial injury in vitro [20, 21].

Thus, this research was designed to investigate the roles and mechanism of oxycodone in mifepristone-induced hEndoSCs during the pathogenesis of endometrial injury. In this report, we hypothesize that mifepristone may induce hEndoSCs injury, oxycodone has a protective effect against endometrial injury in mifepristone-stimulated hEndoSCs, and the latent mechanisms of oxycodone's protective effects may be associated with the inhibition of the TLR4/NF-*κ*B pathway. The findings revealed that oxycodone alleviated endometrial injury by inhibiting the TLR4/NF-*κ*B signaling pathway and could be a novel therapeutic agent for endometrial injury.

## 2. Materials and Methods

### 2.1. Establishment of Endometrial Injury In Vitro Model

The hEndoSCs were brought from the American Type Culture Collection and maintained in DMEM/F-12 medium (Gibco, CA, USA) supplemented with 10% fetal bovine serum (FBS, Gibco), 100 mg/ml streptomycin, and 100 IU/ml penicillin and cultured in a humidified incubator with 5% CO_2_ at 37°C. To establish the endometrial injury in vitro model, hEndoSCs were treated with 60 *μ*mol/l mifepristone (Sigma, USA) at 37°C for 48 h.

### 2.2. MTT Assay

After treatment with different concentrations of oxycodone, hEndoSCs were seeded into 96-well plates at 37°C. Then, the cells were treated with 10 *μ*l MTT (5 mg/ml) solution and continuously incubated for another 4 h. After incubation, the culture medium was removed, and 150 *μ*l DMSO was added to each well to dissolve the formazan product in darkness for 10 min. Finally, the optical density (OD) at the wavelength of 570 nm was measured by a microplate reader (BioTek, USA) after vibration mixing following the manufacturer's instructions.

### 2.3. Flow Cytometry Analysis

After treatment with different concentrations of oxycodone, apoptosis of hEndoSCs was detected by an Annexin V-FITC/PI apoptosis detection kit (Beyotime) following the manufacturer's instructions. Finally, apoptotic cells were measured using the flow cytometer (BD Biosciences, USA) and analyzed using Kaluza analysis software (version 2.1.1.20653; Beckman Coulter, Inc.).

### 2.4. Western Blot Assay

After treatment with different concentrations of oxycodone, the total proteins were harvested from hEndoSCs with RIPA lysis buffer (Beyotime). The protein concentration was valued using a BCA Protein Assay kit (Invitrogen, USA). Then, averaged proteins (40 *μ*g per lane) were resolved by 10% SDS-PAGE and transferred onto PVDF membrane. After blocking with 5% skim milk in PBST for 1 h, the membranes were cultured in primary antibodies against GAPDH (37 kDa; 1 : 1,000; cat. no. 5174; Cell Signaling Technology, Inc.), Bcl-2 (26 kDa; 1 : 1,000; cat. no. 4223; Cell Signaling Technology, Inc.), Bax (20 kDa; 1 : 1,000; cat. no. 5023; Cell Signaling Technology, Inc.), TLR4 (95 kDa; 1 : 1,000; cat. no. sc-2930729; Santa Cruz Biotechnology), p-p65 (65 kDa; 1 : 1,000; cat. no. 3033; Cell Signaling Technology, Inc.), and p65 (65 kDa; 1 : 1,000; cat. no. 8242; Cell Signaling Technology, Inc.) overnight at 4°C. Then, the membranes were treated with an HRP-conjugated secondary antibody (1 : 2,000; cat. no. 7074/7076; Cell Signaling Technology, Inc.) for 1 h. Finally, the protein bands were visualized by ECL detection system reagents (Pierce, USA) and quantified using Image J v.2.0 software (National Institutes of Health).

### 2.5. ELISA

After treatment with different concentrations of oxycodone, the secretion levels of TNF-*α* (cat. no. PT518; Beyotime, Shanghai, China), IL-1*β* (cat. no. PI305; Beyotime, Shanghai, China), and IL-6 (cat. no. PI330; Beyotime, Shanghai, China) in the supernatant of hEndoSCs were detected using ELISA kits according to the manufacturer's protocols. Briefly, hEndoSCs were centrifuged for 5 min at 500 × g, and then, the supernatants were collected, and the levels of TNF-*α*, IL-1*β*, and IL-6 in the supernatant were detected by ELISA.

### 2.6. LDH Assay

LDH released from cells were detected using an LDH-cytotoxicity assay kit (Sigma, USA). Briefly, after treatment with different concentrations of oxycodone, the supernatant of hEndoSCs was collected from each well. Then, the culture supernatant and cell lysates were cultivated with LDH reaction mixture following the manufacturer's protocols for 15 min. The absorbance was detected at 490 nm, and LDH release was calculated with a microplate reader (BioTek, USA).

### 2.7. RT-qPCR Analysis

After treatment with different concentrations of oxycodone, the levels of Bcl-2, Bax, TLR4, p65, or GAPDH were measured by RT-qPCR. The isolation of RNA from hEndoSCs was carried out with the RNA-isolation kit (Life Technologies, USA) following the manufacturer's protocol. Then, the total RNA was reverse transcribed into cDNA in accordance with the instructions of PrimeScript RT Reagent Kit (TaKaRa, China), and qPCR analysis was conducted using the SYBR PrimeScript RT-PCR Kit (TaKaRa) with the ABI 7500 Real-Time PCR System (Agilent Technologies, USA). Target gene expressions were calculated using the 2^−ΔΔCt^ method. Primer sequences for PCR were listed as follows:

  GAPDH: forward, 5′-TCAACGACCACTTTGTCAAGCTCA-3′; reverse, 5′-GCTGGTGGTCCAGGGGTCTTACT-3′;
  Bcl-2: forward, 5′-AGGATTGTGGCCTTCTTTGAG-3′; reverse, 5′-AGCCAGGAGAAATCAAACAGAG-3′;
  Bax: forward, 5′-TCTGAGCAGATCATGAAGACAGG-3′; reverse, 5′-ATCCTCTGCAGCTCCATGTTAC-3′;
  TLR4: forward, 5′-AGACCTGTCCCTGAACCCTAT-3′; reverse, 5′-CGATGGACTTCTAAACCAGCCA-3′;
  p65: forward, 5′-ATGTGGAGATCATTGAGCAGC-3′; reverse, 5′-CCTGGTCCTGTGTAGCCATT-3′.

### 2.8. Statistical Analysis

All results were presented as the means ± SD and analyzed by using GraphPad Prism 6.0. The statistical significant differences among two groups were estimated with unpaired Student's *t*-test, and differences among multiple groups were analyzed using one-way ANOVA. ^*∗*^*P* < 0.05 and ^*∗∗*^*P* < 0.01 were defined as statistically significant.

## 3. Results

### 3.1. Endometrial Injury Model Was Successfully Established by Mifepristone

**Figure 1:**
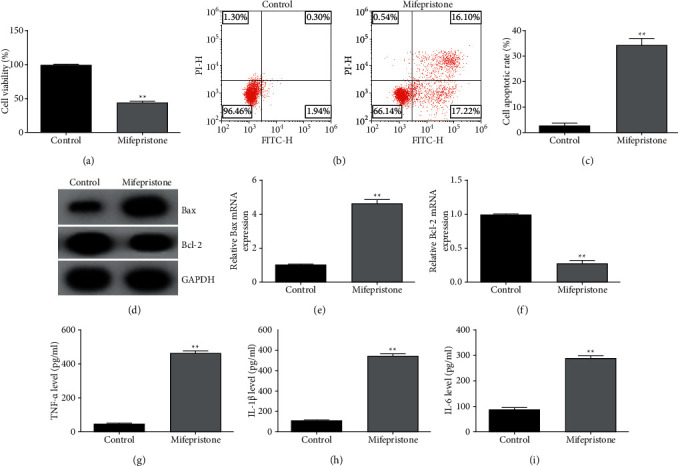
Establishment of endometrial injury model. hEndoSCs were treated with 60 *μ*mol/l mifepristone to generate endometrial injury model in vitro. (a) MTT assay applied to determine cell viability. (b) Apoptotic cells calculated using flow cytometry assay. (c) Quantitative analysis of apoptotic cells. (d–f) Western blot analysis of Bax and Bcl-2 expressions. (h-i) The secretion of inflammatory cytokines (TNF-*α*, IL-1*β*, and IL-6) evaluated using ELISA. All experiments were repeated for three times. ^*∗∗*^*P* < 0.01 vs. control.

hEndoSCs were treated with mifepristone to induce the endometrial injury model in vitro. Then, the cell viability, secretion of inflammatory cytokines, and cell apoptosis were detected to evaluate the endometrial injury. This study found that hEndoSCs viability was notably inhibited in the mifepristone group compared to that of the control group (Figure 1(a)). Besides, mifepristone treatment memorably induced cell apoptosis (Figures 1(b) and 1(c)) and affected the apoptosis-related proteins expression, as evidenced by decreased Bcl-2 and increased Bax (Figures 1(d)–1(f)). In addition, as shown in Figures 1(g)–1(i), the secretion of inflammatory cytokines (TNF-*α*, IL-1*β*, and IL-6) was significantly increased in the mifepristone group, as compared to that of the control group. In summary, the findings indicated that the endometrial injury in vitro model was successfully established by mifepristone.

### 3.2. Oxycodone Had No Cytotoxic Effect on hEndoSCs

**Figure 2:**
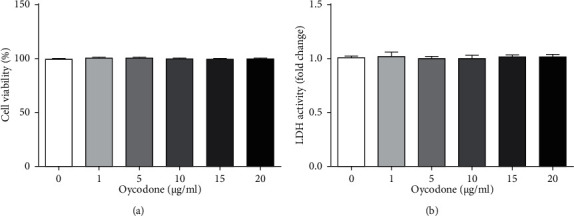
Effects of oxycodone on the viability and LDH release of hEndoSCs. hEndoSCs were induced by 1, 5, 10, 15, or 20 *μ*g/ml oxycodone for 24 h. (a) Cell viability evaluated using MTT assay. (b) LDH activity measured and presented as fold of control. All experiments were repeated for three times.

We illustrated the cytotoxic effect of oxycodone on hEndoSCs. hEndoSCs were induced by 1, 5, 10, 15, and 20 *μ*g/ml oxycodone for 48 h. Then, cells viability and LDH release were evaluated using MTT and LDH assays. The data indicated that no obvious differences were observed in the cell viability and LDH secretion in different groups (Figures 2(a) and 2(b)), indicating that oxycodone had no cytotoxic effect on hEndoSCs.

### 3.3. Protective Effect of Oxycodone on Mifepristone-Induced hEndoSCs Injury

**Figure 3:**
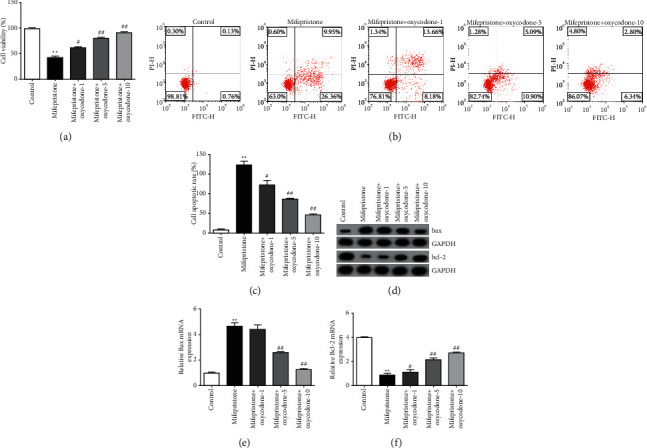
Effects of oxycodone on the mifepristone-induced hEndoSCs injury. hEndoSCs were subjected to 60 *μ*mol/l mifepristone for 48 h and then incubated in 1, 5, or 10 *μ*g/mL oxycodone for 24 h. Cells were divided into five groups: control group, mifepristone group, mifepristone + oxycodone-1 group, mifepristone + oxycodone-5 group, and mifepristone + oxycodone-10 group. (a) Cell viability evaluated using MTT assay. (b) The level of apoptotic hEndoSCs measured using flow cytometry. (c) Quantification of apoptotic cells in different groups. (d–f) Protein and mRNA levels of Bax and Bcl-2 checked using Western blot assay and RT-qPCR. All experiments were repeated for three times. ^*∗∗*^*P* < 0.01 vs. control. ^#^^,^^##^*P* < 0.05, 0.01 vs. the mifepristone treatment group.

To further investigate the roles of oxycodone in mifepristone-triggered hEndoSCs injury, hEndoSCs were exposed to mifepristone for 48 h, followed by treatment with 1, 5, or 10 *μ*g/ml oxycodone for 24 h. hEndoSCs viability and apoptosis were then evaluated using MTT and FCM. As shown in Figures 3(a)–3(c), the viability of hEndoSCs was obviously suppressed, and apoptotic cells were significantly augmented in the mifepristone treatment group compared to that of the control group. This study also determined the apoptosis-related protein expressions, including Bcl-2 and Bax. Results indicated that mifepristone exposure markedly reduced Bcl-2 expression but increased Bax expression levels in mifepristone-induced hEndoSCs (Figures 3(e)–3(f)). However, all these findings were reversed by oxycodone treatment. These findings indicated the protective effect of oxycodone in the mifepristone-mediated hEndoSCs injury.

### 3.4. Oxycodone Reduced the Secretion of Inflammatory Cytokines in Mifepristone-Induced hEndoSCs Injury

**Figure 4:**
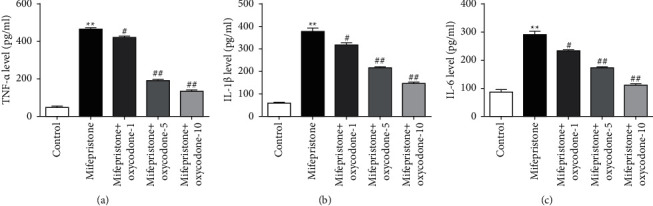
Effects of oxycodone on the mifepristone-induced inflammatory response in hEndoSCs. hEndoSCs were subjected to 60 *μ*mol/l mifepristone for 48 h and then incubated in 1, 5, or 10 *μ*g/mL oxycodone for 24 h. Cells were divided into five groups: control group, mifepristone group, mifepristone + oxycodone-1 group, mifepristone + oxycodone-5 group, and mifepristone + oxycodone-10 group. ELISA assay was conducted to evaluate TNF-*α*, IL-1*β*, and IL-6. (a–c) Expression in different groups. All experiments were repeated for three times. ^*∗∗*^*P* < 0.01 vs. control. ^#^^,^^##^*P* < 0.05, 0.01 vs. the mifepristone treatment group.

To investigate whether the inflammatory cytokines, TNF-*α*, IL-1*β*, and IL-6, were affected by oxycodone treatment, ELISA was performed. As shown in Figures 4(a)–4(c), compared with the control group, the secretion of inflammatory cytokines (TNF-*α*, IL-1*β*, and IL-6) in the supernatant of hEndoSCs was significantly increased after mifepristone treatment. As expected, oxycodone decreased the secretion of inflammatory cytokines in the supernatant of hEndoSCs in dose-dependent manner. The findings demonstrated that oxycodone reduced inflammatory factors production in mifepristone-induced hEndoSCs damage.

### 3.5. Oxycodone Inhibited the Activation of TLR4/NF-*κ*B Signaling Pathway in Mifepristone-Induced hEndoSCs Injury

**Figure 5:**
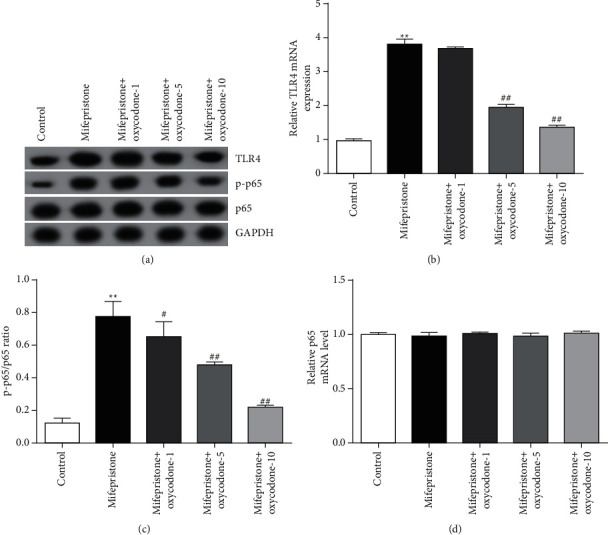
Effects of oxycodone on the TLR4/NF-*κ*B signal pathway in mifepristone-induced hEndoSCs. After treated with mifepristone, hEndoSCs were incubated in different concentrations of oxycodone for 24 h. (a) Protein expression of TLR4 and abundance of p-p65 determined by Western blot assay. (b) mRNA levels of TLR4 determined by RT-qPCR analysis. (c) Quantification of p-p65/p65 ratio expressed. (d) mRNA levels of p65 in different groups measured using RT-qPCR analysis. All experiments were repeated for three times. ^*∗∗*^*P* < 0.01 vs. control. ^#^^,^^##^*P* < 0.05, 0.01 vs. the mifepristone treatment group.

Finally, we illustrated the latent mechanism of oxycodone in mifepristone-induced hEndoSCs. Western blot assay and RT-qPCR analysis indicated that mifepristone treatment obviously increased the expressions of TLR4; nevertheless, oxycodone significantly reversed the effects of mifepristone on the level of TLR4 (Figures 5(a) and 5(b)). Western blot assay revealed that mifepristone treatment significantly enhanced the level of p-p65 and p-p65/p65 ratio (Figures 5(a) and 5(c)). Besides, these promotions in p-p65 abundance and p-p65/p65 ratio levels were reversed by oxycodone treatment (Figures 5(a) and 5(c)). Meanwhile, there was no significant difference in p65 mRNA levels among all groups (Figure 5(d)). Collectively, the findings suggested that TLR4/NF-*κ*B signaling was related to the mifepristone-induced hEndoSCs, and oxycodone exhibited a protective role in mifepristone-induced hEndoSCs injury via inhibiting the TLR4/NF-*κ*B pathway (Supplementary Figure 1).

## 4. Discussion

Oxycodone, a semisynthetic opioid analgesic, has high bioavailability. Clinically, oxycodone is often used to treat cancer pain or painless egg extraction. Deng et al. suggested that Arl6ip-1 may be a candidate target in the cancer-induced bone pain rat model after oxycodone treatment [22]. Meanwhile, Yang et al. revealed the effects of oxycodone on treatment of neuropathic pain in mice [23]. Endometrial injury is a common gynecological disease, caused by serious endocrine disorders, artificial abortion, inflammation, infection, and other factors, and severe endometrial injury may cause infertility [4, 24]. However, the specific pathogenesis in endometrial injury has not been completely illuminated. Therefore, it is of great clinical significance to find an effective treatment for endometrial injury. This study focus on the investigation of the effects of oxycodone on endometrial injury.

According to previous research studies, mifepristone [21], curettage, or coagulation was applied to establish endometrial injury models [25]. Mifepristone is often used to soften the cervix after a miscarriage and can also be used in emergency contraception [26]. In this study, hEndoSCs were exposed to mifepristone to induce endometrial injury model in vitro. Then, the cell viability, LDH release, cell apoptosis, and secretion of inflammatory cytokines were detected to evaluate whether the in vitro model of endometrial injury is successfully established. Consistent with previous research [21], this study indicated that mifepristone treatment resulted in inhibited hEndoSCs viability, enhanced cell apoptosis, decreased Bcl-2 expression, increased Bax expression, and promoted inflammatory cytokines secretion, indicating that the endometrial injury in vitro model was successfully established.

To evaluate the effects of oxycodone on mifepristone-induced endometrial injury, this research first evaluated the cytotoxic effect of oxycodone on hEndoSCs, and the findings indicated that oxycodone had no cytotoxic effect on hEndoSCs. To further explore the effects of oxycodone on mifepristone-triggered endometrial injury, mifepristone-induced hEndoSCs were treated with 1, 5, or 10 *μ*g/ml oxycodone for 24 h. The data indicated the protective effect of oxycodone on mifepristone-induced endometrial injury, as evidenced by promoted cell viability and suppressed apoptotic cells. It should be noted that this study did not provide the cell morphology of each group of hEndoSCs to show the damage model and health status of the cells under different treatments. This was a limitation of this study.

Besides, it is well known that cell apoptosis usually triggered endogenic apoptotic cascades through Bcl-2 family regulators [27]. The proapoptotic proteins such as Bax may induce cell death, and the antiapoptotic protein Bcl-2 could promote cell survival [28]. This study also determined the expressions of Bcl-2 and Bax in mifepristone-treated hEndoSCs. The data of the present study suggested that oxycodone exposure markedly increased Bcl-2 expression, but reduced Bax expression levels in mifepristone-treated hEndoSCs, demonstrating the protective effect of oxycodone in the mifepristone-mediated hEndoSCs injury.

It has been proposed that inflammatory cytokines such as IL-1*β* and IL-6 contribute to the establishment of endometrial injury [29, 30]. The present study also evaluated the releases of TNF-*α*, IL-1*β*, and IL-6 in the supernatants of hEndoSCs. Results from ELISA suggested that oxycodone decreased the secretion of inflammatory cytokines in mifepristone-induced hEndoSCs in a dose-dependent manner, demonstrating that oxycodone inhibited inflammatory response in mifepristone-induced hEndoSCs damage. What needs to be mentioned is that the mechanism of IL-1*β* release has proven to be elusive, and it does not follow the conventional ER-Golgi route of secretion [31]. In this study, we did not further explore the secretion mechanism of IL-1*β* in mifepristone-induced hEndoSCs. This was another limitation of this study, and we will explore this in depth in the next research.

Numerous reports have approved that multiple pathways were related to pathogenesis of endometrial injury, containing the mitogen-activated protein kinase (MAPK) signal pathway and NF-*κ*B signal pathway [20, 32]. NF-*κ*B was first identified as a transcription factor in the B lymphocyte nucleus that mediates gene transcription by binding to specific sequences [33]. The activation of NF-*κ*B results in transcription of many proinflammatory genes and upregulation of cytokines and chemokines [34]. To better investigate the latent mechanism of the effect of oxycodone on mifepristone-triggered endometrial injury, the TLR4/NF-*κ*B pathway was explored. As expected, mifepristone treatment obviously increased the expressions of TLR4 and enhanced the abundance of p-p65 and p-p65/p65 ratio, while these promotions were reversed by oxycodone treatment. These data suggested that the protective effects of oxycodone in mifepristone-induced hEndoSCs might be regulated, at least partly, via inhibiting the TLR4/NF-*κ*B signaling pathway.

However, this study is only a preliminary in vitro study of the effect of oxycodone on endometrial injury. To clarify the role and molecular mechanism of oxycodone in endometrial injury, more in-depth studies are needed. For example, whether IL-1*β* section is related to ROS/NLRP3 signaling needs to be explored. Besides, which ligand activated TLR4 in mifepristone-triggered hEndoSCs should be further clarified. In addition, the effect of oxycodone on endometrial injury should be studied in animal models. We will perform these issues in the next research.

In conclusion, this research revealed that oxycodone exhibited the protective effects on mifepristone-triggered endometrial injury through the inhibition of TLR4/NF-*κ*B signal pathway, which might be as a promising candidate for endometrial injury treatment in clinics.

## Data Availability

All datasets used and/or generated during the current study are available from the corresponding author upon request.

## Supplementary Materials

Supplementary Materials Supplementary Figure 1. Schematic diagram illustrating the effect of oxycodone on mifepristone-induced hEndoSCs through the TLR4/NF-*κ*B pathway.

## References

[1] Xin L., Lin X., Zhou F. (2020). A scaffold laden with mesenchymal stem cell-derived exosomes for promoting endometrium regeneration and fertility restoration through macrophage immunomodulation. *Acta Biomaterialia*.

[2] Devesa-Peiro A., Sebastian-Leon P., Garcia-Garcia F. (2020). Uterine disorders affecting female fertility: what are the molecular functions altered in endometrium?. *Fertility and Sterility*.

[3] Guney M., Oral B., Demirin H., Karahan N., Mungan T., Delibas N. (2007). Protective effects of vitamins C and E against endometrial damage and oxidative stress in fluoride intoxication. *Clinical and Experimental Pharmacology and Physiology*.

[4] Hilton J., Liu K. E., Laskin C. A., Havelock J. (2019). Effect of endometrial injury on in vitro fertilization pregnancy rates: a randomized, multicentre study. *Archives of Gynecology and Obstetrics*.

[5] Lensen S. F., Manders M., Nastri C. O. (2016). Endometrial injury for pregnancy following sexual intercourse or intrauterine insemination. *Cochrane Database of Systematic Reviews*.

[6] Kinnunen M., Piirainen P., Kokki H., Lammi P., Kokki M. (2019). Updated clinical pharmacokinetics and pharmacodynamics of oxycodone. *Clinical Pharmacokinetics*.

[7] Scheidel B., Maritz M. A., Gschwind Y. J. (2017). Bioavailability of oxycodone after administration of a new prolonged-release once-daily tablet formulation in healthy subjects, in comparison to an established twice-daily tablet. *International Journal of Clinical Pharmacology and Therapeutics*.

[8] Babalonis S., Coe M. A., Nuzzo P. A. (2021). Acute administration of oxycodone, alcohol, and their combination on simulated driving-preliminary outcomes in healthy adults. *Psychopharmacology*.

[9] Raff M., Belbachir A., El-Tallawy S. (2019). Intravenous oxycodone versus other intravenous strong opioids for acute postoperative pain control: a systematic review of randomized controlled trials. *Pain and Therapy*.

[10] Yang Y., Liu P. Y., Bao W., Chen S. J., Wu F. S., Zhu P. Y. (2020). Hydrogen inhibits endometrial cancer growth via a ROS/NLRP3/caspase-1/GSDMD-mediated pyroptotic pathway. *BMC Cancer*.

[11] Zhang L., Wan Y., Zhang Z. (2020). FTO demethylates m6A modifications in HOXB13 mRNA and promotes endometrial cancer metastasis by activating the WNT signalling pathway. *RNA Biology*.

[12] Xu Q., Ge Q., Zhou Y. (2020). MELK promotes Endometrial carcinoma progression via activating mTOR signaling pathway. *EBioMedicine*.

[13] Bai Y., Li Z., Liu W., Gao D., Liu M., Zhang P. (2019). Biochanin A attenuates myocardial ischemia/reperfusion injury through the TLR4/NF-*κ*B/NLRP3 signaling pathway. *Acta Cirurgica Brasileira*.

[14] Zhou W., Chen X., Hu Q., Chen X., Chen Y., Huang L. (2018). Galectin-3 activates TLR4/NF-*κ*B signaling to promote lung adenocarcinoma cell proliferation through activating lncRNA-NEAT1 expression. *BMC Cancer*.

[15] Cadepond F., Ulmann A., Baulieu E.-E. (1997). RU486 (mifepristone): mechanisms of action and clinical uses. *Annual Review of Medicine*.

[16] Boggavarapu N. R., Berger C., von Grothusen C., Menezes J., Gemzell-Danielsson K., Lalitkumar P. G. L. (2016). Effects of low doses of mifepristone on human embryo implantation process in a three-dimensional human endometrial in vitro co-culture system. *Contraception*.

[17] Chen J. G., Chen T., Ding Y. (2015). Baicalin can attenuate the inhibitory effects of mifepristone on Wnt pathway during peri-implantation period in mice. *The Journal of Steroid Biochemistry and Molecular Biology*.

[18] Cho Y. J., Park S. B., Han M. (2015). Di-(2-ethylhexyl)-phthalate induces oxidative stress in human endometrial stromal cells *in vitro*. *Molecular and Cellular Endocrinology*.

[19] Li Y., Lin Y., Huang X. (2019). SCM-198 protects endometrial stromal cells from oxidative damage through Bax/Bcl-2 and ERK signaling pathways. *Acta Biochimica et Biophysica Sinica*.

[20] Zhu H., Jiang Y., Pan Y., Shi L., Zhang S. (2018). Human menstrual blood-derived stem cells promote the repair of impaired endometrial stromal cells by activating the p38 MAPK and AKT signaling pathways. *Reproductive Biology*.

[21] Wang J., Hu R., Xing Q. (2020). Exosomes derived from umbilical cord mesenchymal stem cells alleviate mifepristone-induced human endometrial stromal cell injury. *Stem Cells International*.

[22] Deng H.-S., Xu L.-S., Ni H.-D. (2019). Proteomic profiling reveals Arl6ip-1 as a candidate target in cancer-induced bone pain rat model after oxycodone treatment. *Neuroscience Letters*.

[23] Yang P.-P., Yeh G.-C., Huang E. Y.-K., Law P.-Y., Loh H. H., Tao P.-L. (2015). Effects of dextromethorphan and oxycodone on treatment of neuropathic pain in mice. *Journal of Biomedical Science*.

[24] Xia L., Meng Q., Xi J. (2019). The synergistic effect of electroacupuncture and bone mesenchymal stem cell transplantation on repairing thin endometrial injury in rats. *Stem Cell Research & Therapy*.

[25] Yanpeng W., Qiongxiao H., Sheng X. U., Jing S. (2017). Establishment of mouse endometrial injury model by curettage or coagulation. *Zhejiang Da Xue Xue Bao Yi Xue Ban*.

[26] Rodriguez M. I., Gülmezoglu A. M. (2020). Mifepristone for emergency contraception: case for recommendation in practice guidelines. *Contraception*.

[27] Luo C., Zhu Y., Jiang T. (2007). Matrine induced gastric cancer MKN45 cells apoptosis via increasing pro-apoptotic molecules of Bcl-2 family. *Toxicology*.

[28] Kim E. M., Jung C.-H., Song J.-Y., Park J. K., Um H.-D. (2018). Pro-apoptotic Bax promotes mesenchymal-epithelial transition by binding to respiratory complex-I and antagonizing the malignant actions of pro-survival Bcl-2 proteins. *Cancer Letters*.

[29] Lu W., He F., Lin Z. (2021). Dysbiosis of the endometrial microbiota and its association with inflammatory cytokines in endometrial cancer. *International Journal of Cancer*.

[30] Tu Y.-A., Chou C.-H., Yang P.-K. (2021). Intentional endometrial injury enhances angiogenesis through increased production and activation of MMP-9 by TNF-*α* and MMP-3 in a mouse model. *Molecular Human Reproduction*.

[31] Lopez-Castejon G., Brough D. (2011). Understanding the mechanism of IL-1*β* secretion. *Cytokine & Growth Factor Reviews*.

[32] Cui L., Zheng Y., Wang H. (2020). Cortisol inhibits the Escherichia coli-induced endometrial inflammatory response through NF-*κ*B and MAPK pathways in postpartum goats. *Animal Reproduction Science*.

[33] Liu Q., Wang C., Meng Q. (2021). Puerarin sensitized K562/ADR cells by inhibiting NF-*κ*B pathway and inducing autophagy. *Phytotherapy Research*.

[34] Chang R.-J., Wang H.-L., Qin M.-B. (2021). Ghrelin inhibits IKK*β*/NF-*κ*B activation and reduces pro-inflammatory cytokine production in pancreatic acinar AR42J cells treated with cerulein. *Hepatobiliary and Pancreatic Diseases International*.

