# Supplementary material for: Oxycodone Alleviates Endometrial Injury via the TLR4/NF-κB Pathway
Source: Evid Based Complement Alternat Med. 2022 Feb 23;2022:6153279. doi: 10.1155/2022/6153279 (PMC8933090; doi:10.1155/2022/6153279)
Supplement: Supplementary Materials — Supplementary Figure 1. Schematic diagram illustrating the effect of oxycodone on mifepristone-induced hEndoSCs through the TLR4/NF-κB pathway. [file 6153279.f1.pdf]

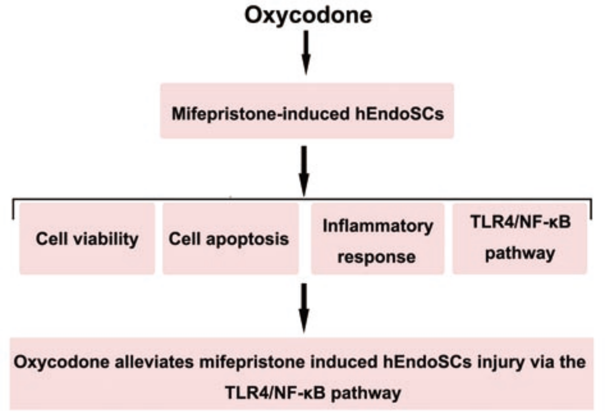
Oxycodone
Mifepristone-induced hEndoSCs
Cell viability
Cell apoptosis
Inflammatory response
TLR4/NF-κB pathway
Oxycodone alleviates mifepristone induced hEndoSCs injury via the TLR4/NF-κB pathway
